# Deletion of caspase-8 in mouse myeloid cells blocks microglia pro-inflammatory activation and confers protection in MPTP neurodegeneration model

**DOI:** 10.18632/aging.100805

**Published:** 2015-09-23

**Authors:** Edel Kavanagh, Miguel Angel Burguillos, Alejandro Carrillo-Jimenez, María José Oliva-Martin, Martiniano Santiago, Johanna Rodhe, Bertrand Joseph, Jose Luis Venero

**Affiliations:** ^1^ Department of Oncology-Pathology, Cancer Centrum Karolinska, R8:03, Karolinska Institutet, 171 76 Stockholm, Sweden; ^2^ Departamento de Bioquímica y Biología Molecular, Facultad de Farmacia, Universidad de Sevilla, and Instituto de Biomedicina de Sevilla (IBiS)-Hospital Universitario Virgen del Rocío/CSIC/Universidad de Sevilla, 41013-Sevilla, Spain

**Keywords:** microglia, Caspace-8, Parkinson's disease, lipopolysaccharide, MPTP

## Abstract

Increasing evidence involves sustained pro-inflammatory microglia activation in the pathogenesis of different neurodegenerative diseases, particularly Parkinson's disease (PD). We recently uncovered a completely novel and unexpected role for caspase-8 and its downstream substrates caspase-3/7 in the control of microglia activation and associated neurotoxicity to dopaminergic cells. To demonstrate the genetic evidence, mice bearing a floxed allele of *CASP8* were crossed onto a transgenic line expressing Cre under the control of *Lysozyme 2* gene. Analysis of caspase-8 gene deletion in brain microglia demonstrated a high efficiency in activated but not in resident microglia. Mice were challenged with lipopolysaccharide, a potent inducer of microglia activation, or with MPTP, which promotes specific dopaminergic cell damage and consequent reactive microgliosis. In neither of these models, *CASP8* deletion appeared to affect the overall number of microglia expressing the pan specific microglia marker, Iba1. In contrast, CD16/CD32 expression, a microglial pro-inflammatory marker, was found to be negatively affected upon *CASP8* deletion. Expression of additional proinflammatory markers were also found to be reduced in response to lipopolysaccharide. Of importance, reduced pro-inflammatory microglia activation was accompanied by a significant protection of the nigro-striatal dopaminergic system in the MPTP mouse model of PD.

## INTRODUCTION

Activated microglia play key roles in neuro-inflammation and depending on the nature of the initial stimulus their actions may be either beneficial or detrimental to neuronal function. In the latter case, activated microglia undergo phenotypic polarization toward a so called M1 pro-inflammatory phenotype and produce neurotoxic factors, such as inflammatory cytokines, reactive oxygen species, nitric oxide synthase (NOS), and glutamate [1, 2]. In contrast, activated microglia can be polarized into an anti-inflammatory phenotype defined as M2 phenotype expressing anti-inflammatory cytokines, growth factors, and unique membrane receptors [3, 4].

Chronic neuroinflammation involves the sustained activation of microglia, and consequent release of proinflammatory mediators, which are detrimental for the neuronal cell population. In chronic neuro-degenerative diseases, microglia remain activated for an extended period during which the production of mediators is sustained longer than usual, thus contributing to neuronal death. Compelling evidence suggests that inflammation plays an important role in the cell loss observed in Parkinson's disease (PD) [5–7]. Epidemiological studies have demonstrated that incidence of idiopathic PD is about 50% lower in chronic users of non-steroidal anti-inflammatory drugs or cyclo-oxygenase inhibitors than in age-matched non-users [8, 9]. Inflammation has been shown in different animal models of PD. Among them, the acute 1-methyl-4-phenyl-1,2,3,6-tetrahydropyridine (MPTP) model has been demonstrated to encompass a significant inflammatory response in the SN pars compacta (SNPC) along with degeneration of the nigro-striatal dopaminergic system [10–12]. LPS is the active immunostimulant in the cell wall of Gram-negative bacteria that is responsible for triggering the cascade of events following bacterial infection [13, 14]. Interestingly, the SN is especially susceptible to the LPS-induced neurotoxicity as compared with other brain areas [15]. In this structure, the strong microglial response to LPS preceded the death of dopaminergic neurons [16, 17].

Caspases, a family of cysteinyl aspartate-specific proteases, are best known as executioners of apoptotic cell death and their activation are considered as a commitment to cell death ( [18]. However, certain caspases also function as regulatory molecules for immunity, cell differentiation and cell-fate determination [19].

We have described an unexpected novel function for caspases in the control of microglia proinflammatory activation and thereby neurotoxicity [20]. We showed that the orderly activation of caspase-8 and caspase-3/7, known executioners of apoptotic cell death, regulates microglia activation. We found that stimulation of microglia with various inflammogens activates caspase-8 and caspase-3/7 in absence of cell death *in vitro* and *in vivo*. Importantly, we also observed that these caspases are activated in microglia in the substantia nigra in PD subjects [20].

We aim at providing the genetic evidence that targeting *CASP8* gene selectively in the microglia cell population in the brain can provide neuronal beneficial effect in animal models of PD. We have taken advantage of conditional knock-out genetic approaches in the myeloid system, including microglia in the brain, to examine cell-specific effects of caspase-8 signaling in the intranigral LPS model and the acute MPTP model of Parkinson's disease, well-established paradigms of dopaminergic neurodegeneration that induces a robust inflammatory response in the substantia nigra. In these models, we confirmed *in vivo* the importance of caspase-8 in regulating the proinflammatory response of activated microglia.

## RESULTS

### Generation of conditional caspase-8 knockout mice in myeloid cells and evaluation of caspase-8 gene deletion in resident and activated microglia

**Figure 1:**
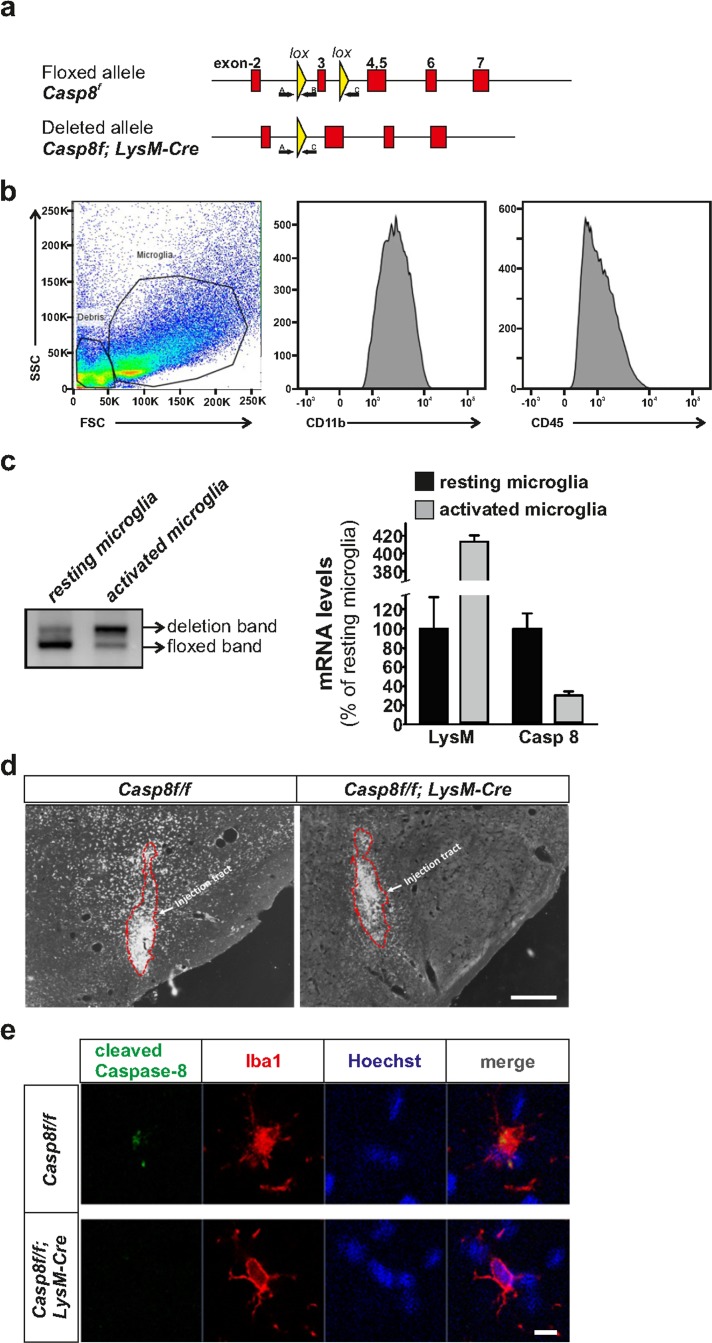
Generation of *Cre^LysM^Casp8^fl/fl^* mice and validation of Caspase-8 deletion Panel (**a**) contains a schematic illustration of the mouse genomic locus showing the insertion of the targeted allele flanked with LoxP sites (upper), and the deleted allele after Cre recombination (lower). Locations of primers A, B and C used for the PCR deletion study are also shown. Flow cytometry demonstrating the purity of the microglial fraction (**b**) used to assess the degree of caspase-8 gene deletion (**c**) based on an ABC primer strategy. The degree of gene caspase-8 gene deletion was evaluated in mesencephalic resident and activated microglia. QPCR demonstrated a low caspase-8 gene deletion in resident microglia and a dramatic increase in activated microglia. mRNA analysis of Lysozyme 2 and caspase-8 in microglia isolated from the ventral mesencephalon from *Cre^LysM^Casp8^fl/fl^* mice corroborated the striking differences between resident and activated microglia (**c**). Panel (**d**) shows immunohistochemistry of cleaved caspase-8 in the ventral mesencephalon of *Casp8^fl/fl^* and *Cre^LysM^Casp8^fl/fl^* mice after intranigral LPS injection. Panel (**e**) shows immunofluorescence of cleaved caspase-8 and Iba1 showing co-staining in MPTP-injected *Casp8^fl/fl^* mice, and absence of cleaved caspase-8 staining in *Cre^LysM^Casp8^fl/fl^* mice (**f**). Scale bar: e: 300 μm; f: 20 μm.

Caspase-8 floxed animals (*Casp8^fl/fl^*) expressing Lysoxyme 2 (LysM) Cre will be referred to as *Cre^LysM^Casp8^fl/fl^*. Analysis of caspase-8 staining in the substantia nigra after LPS intranigral injection revealed a significant decrease in cleaved caspase-8 in *Cre^LysM^Casp8^fl/fl^* mice (Fig. 1d,e). We performed dual caspase-8 and Iba1 immunofluorescence after either intranigral LPS injection or MPTP administration. Cleaved caspase-8 immuofluorescence was detected in Iba1-positive cells of *Casp8^fl/fl^* mice after stimulation with either LPS or MPTP. Reduced cleaved caspase-8 staining was observed in Iba1-positive cells of *Cre^LysM^Casp8^fl/fl^* mice, confirming the depletion of caspase-8 in brain myeloid cells (Fig. 1e). Some concerns have been recently raised concerning the use of *Cre^LysM^* mice to specifically target adult microglia [21]. Previous studies reported a high rate of recombination in brain microglia when using these mice [22, 23]. In contrast, an average recombination rate of about 45% was obtained in Iba1-labelled resident microglia from *Cre^LysM^* mice. However, our immunohistochemical analysis demonstrated an almost absence of cleaved caspase-8 immunoreactivity in reactive microglia, suggesting an effective deletion of *Caspase-8* gene in those cells in the *Cre^LysM^* mice. Ascertaining the rate of *Caspase-8* gene deletion in our experimental conditions is of critical importance considering the modest degree of recombination reported by Goldmann et al [21]using *Cre^LysM^* mice. We wondered whether resting and activated microglia could exhibit different rates of gene deletion in the *Cre^LysM^* mice. To achieve that, we isolated microglia from the ventral mesencephalon from intact and LPS-injected mice. Microglia were immunopurified using CD-11b-labelled microbeads after myelin removal by Percoll treatment. The purity of the microglia preparation was evaluated by flow cytometry analysis using CD-11b and CD-45 antibodies (Fig. 1b). Genomic DNA was isolated from resting and activated microglia and deletion of the *Caspase-8* floxed sequence was evaluated following an ABC primer strategy. While primers A and B detect the floxed sequence, primers A and C do not amplify unless the floxed sequence is deleted (Fig. 1a). We have recently developed mice in which one of the caspase-8 alleles was knocked out (50% deletion). Microglia isolated from these mice was used to normalize the QPCR analysis. By using this approach, resident microglia from *Cre^LysM^Casp8^fl/fl^* mice rendered an extremely low level of *Caspase-8* gene deletion (7.1 ± 3.2%), a level even lower than that reported by Goldmann et al [21]. In contrast, activated microglia obtained from the ventral mesencephalon from LPS-injected animals showed a very high rate of *Caspase-8* gene deletion (75.4 ± 10.2) (Figure 1). A similar approach has been previously employed by Cho and coworker [24]. Indeed, the authors stimulated *Cre^LysM^IKKβ^f/f^* mice *in vivo* by *i.c.v* LPS injection and kainic acid injection, and demonstrated that the deletion rate is notably increased upon microglia activation. Furthermore, the authors demonstrated significantly less proinflammatory microglia activation in the mice subjected to kainic acid treatment [24]. To further validate the efficiency of Caspase-8 gene deletion, we analyzed the mRNA levels of Lysozyme M and caspase-8 from microglia isolated from the ventral mesencephalon 24 h after intranigral LPS injections. As seen in Fig. 1c, Lysozyme M mRNA levels were robustly up-regulated in activated microglia. Under these conditions, caspase-8 mRNA levels dropped to 30% of levels of resident microglia obtained from *C Cre^LysM^Casp8^fl/fl^ re^LysM^Casp8^fl/fl^ Cre^LysM^Casp8^fl/fl^* mice. Due to tissue dissecting limitations, it is reasonable to assume that not all microglia isolated from the ventral mesencephalon from LPS-injected animals are activated. Consequently, we may conclude that *Cre^LysM^*mice are valid to target adult microglia under experimental conditions leading to proinflammatory activation.

### Caspase-8 deletion in myeloid cells blocks microglia activation in LPS neuroinflammatory model

As a first approach, we used intranigral injections of LPS, a major endotoxin acting as a ligand of TLR4 and known to induce a classically proinflammatory activation of microglia. Intranigral injection of LPS has been shown to induce an extensive activation of microglia along with degeneration of substantia nigra dopaminergic neurons [16, 25].

#### Immunohistochemistry analysis of microglia/macro-phage population

**Figure 2:**
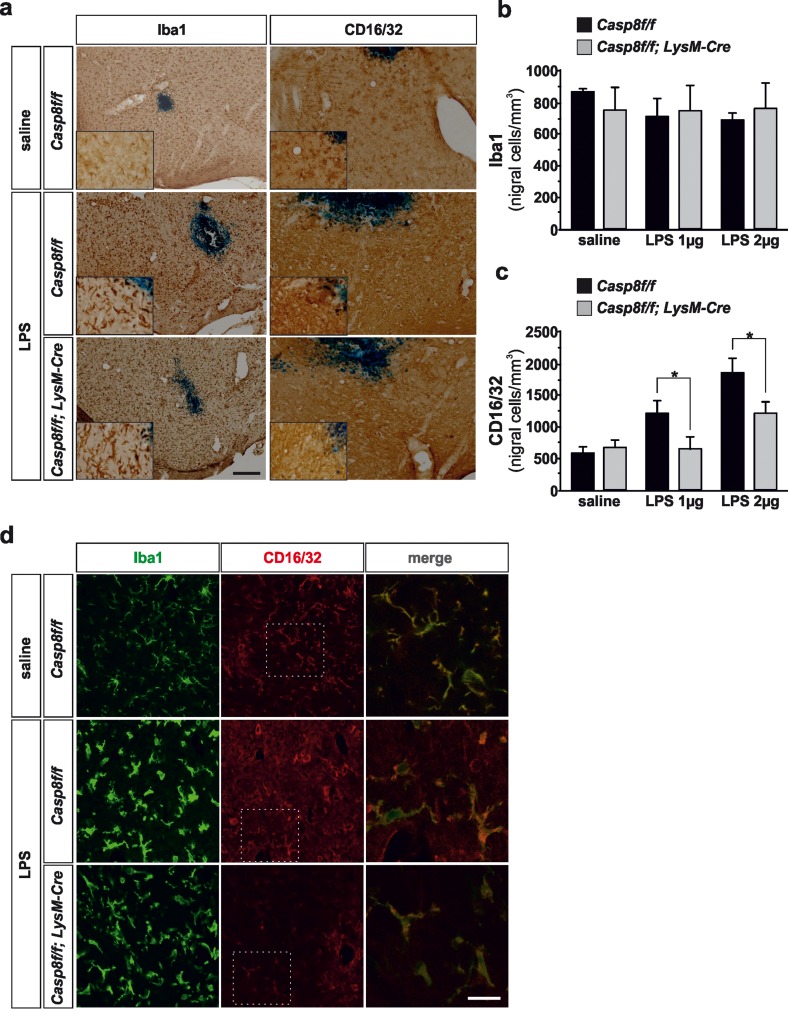
Microglial caspase-8 deficiency ameliorates proinflammatory microglia activation in the substantia nigra in response to intranigral LPS injection Panel (**a**) shows an illustration of Iba1 and CD16/32-labeled microglia in the ventral mesencephalon in response to either saline or intranigral LPS in *Casp8^fl/fl^* mice and *Cre^LysM^Casp8^fl/fl^* mice. The effect of LPS and caspase-8 deficiency on the number of total microglia (Iba1) or proinflammatory microglia (CD16/32) in the substantia nigra is shown in panels (**b**) and (**c**). Results are the mean ± SD of a minimum of four independent experiments and are expressed as number of cells per mm^2^. Statistical significance was calculated by analysis of variance followed by the least significant difference *post hoc* test for multiple range comparisons (*p* <0.05). Panel (**d**) shows an illustration of dual immunofluorescence of Iba1 and CD16/32-labeled microglia in the ventral mesencephalon in response to saline or intranigral LPS in *Casp8^fl/fl^* mice and *Cre^LysM^Casp8^fl/fl^* mice. Merge photographs shown in (**d**) are higher magnification photographs of dot boxes depicted in the left column. Note the completely different morphological features of microglia in response to saline or LPS (**d**). No apparent differences in terms of Iba1 were detected between *Casp8^fl/fl^* mice and *Cre^LysM^Casp8^fl/fl^* mice (a, b, d). Also note the specific labeling of CD16/32, a proinflammatory marker of microglia activation, which is located in the membrane of microglia (d). Also note how the expression of CD16/32 is clearly down-regulated in response to caspase-8 deficiency (**a, c, d**). Scale bar: a, 100 μm; d: Iba1 and CD16/32 staining: 50 μm; merge: 20 μm.

**Figure 3:**
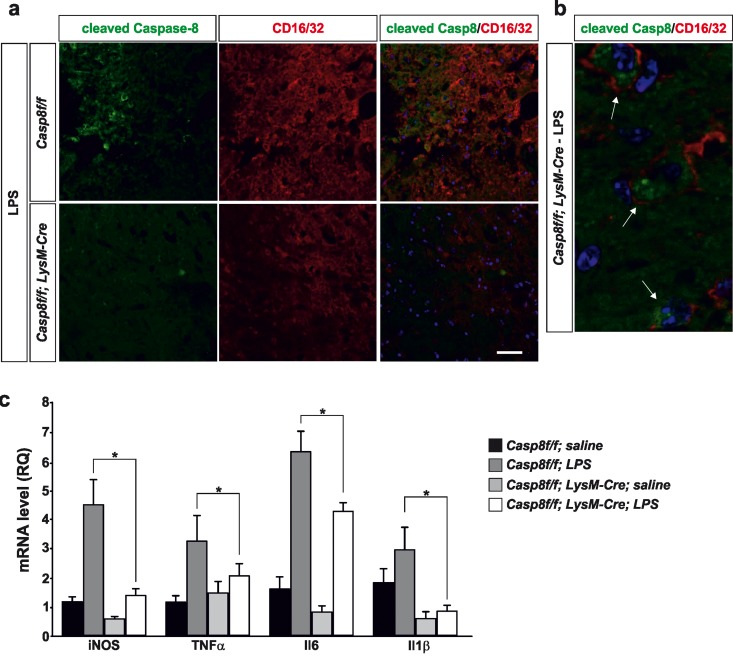
Caspase-8 is activated in viable CD16/32-immunolabeled microglia in substantia nigra in response to intranigral LPS injection, which is associated to higher key proinflammatory microglial markers Panel (**a**) shows an illustration of dual immunofluorescence of cleaved caspase-8 and CD16/32 in substantia nigra in response to intranigral LPS in *Casp8^fl/fl^* mice and *Cre^LysM^Casp8^fl/fl^* mice. Note how cleaved caspase-8 labeling is mostly associated to CD16/32-labeled microglia, which is dramatically decreased in *Cre^LysM^Casp8^fl/fl^* mice. Panel (**b**) shows higher magnification photograph of that shown in panel (**a**) demonstrating active caspase-8 within viable CD16/32-immunolabeled microglia in *Casp8^fl/fl^* mice after LPS. Panel (**c**) shows the effect of LPS on mRNA expression of inducible nitric oxide synthase (iNOS), tumour necrosis factor α (TNF- α), interleukin 6 (IL-6) and interleukin-1β in substantia nigra of *Casp8^fl/fl^* mice and *Cre^LysM^Casp8^fl/fl^* mice. mRNA levels were measured by quantitative PCR. Results are mean ± SD of at least four independent experiments and are expressed as relative quantification (RQ), calculated using the delta Ct method. Statistical significance was calculated by one-way analysis of variance followed by the least significant difference *post hoc* test for multiple range (*p* <0.05). As expected, LPS injection increased the expression levels of mRNA in *Casp8^fl/fl^* mice (WT). This induction was highly prevented in *Cre^LysM^Casp8^fl/fl^* mice (KO). Note the extremely low levels of IL-1β in *Cre^LysM^Casp8^fl/fl^* mice. Scale bar: a: 30 μm; b: 10 μm.

Tissues were immunostained with Iba1, a pan specific marker of microglia/macrophages and CD16/32, a specific marker for the proinflammatory phenotype. We analyzed the effect of two *in vivo* doses of LPS, 2 and 4 μgs. Caspase-8 inhibition in LPS-induced activated microglia has been shown to induce necroptosis [26, 27], a RIPK1/RIPK3-dependent process [28, 29]. Analysis of microglia using Iba1 did not show differences in *Cre Cre^LysM^Casp8^fl/fl Lys^MCasp8^fl/fl^* mice as compared with *Casp8^fl/fl^* mice in response to LPS (Fig. 2a, b), an indication that necroptosis was not occurring in our experimental conditions. Upon LPS treatment, there was a marked microglia morphological change as characterized by Iba1 reactivity; further thickening of processes, retraction of finer ones, increased cell body size and Iba1 reactivity (Fig. 2a, d). In addition, numerous amoeboid-shaped cells could be observed in LPS-injected animals in both Casp8^fl/fl^ mice and *Cre^LysM^Casp8^fl/fl^* mice. Since Iba1 does not clearly discern between resident and activated microglia, we used a specific marker for the proinflammatory phenotype of microglia, CD16/32 [30–32]. CD16/32 was significantly up-regulated in Iba-1-labeled microglia from *Casp8^fl/fl^* mice (Fig. 2d). However, this LPS-induced up-regulation of microglial CD16/32 was significantly attenuated in *Cre^LysM^Casp8^fl/fl^* mice (Figs. 2a, b, d and 3a). Dual immunofluorescence of Iba1 and CD16/32 demonstrated that upon saline injection there was some degree of microglia activation characterized by increased ramification of cytoplasmic process and cell size and enhanced Iba1 labeling (Fig. 2d). Under these conditions, most if not all CD16/32 positive cells were also positive for Iba1 (Fig. 2d).

#### Analysis of cleaved caspase-8 in proinflammatory microglia after LPS

We then analyzed the extent of active cleaved caspase-8 in activated microglia in the ventral mesencephalon in response to LPS injection. We found a widespread distribution of cleaved caspase-8 cells in LPS-injected *Casp8^fl/fl^* mice (Fig. 3a, b). In *Cre^LysM^Casp8^fl/fl^* mice, the labeling of cleaved caspase-8 was extremely low (Fig. 3a). Considering that we used conditional *CASP8* knock out mice, our data suggest that under conditions of proinflammatory microglia activation, there is a selective activation of caspase-8 within microglia/macrophages. This observation is in line with our previous data demonstrating selective activation of caspase-8 in reactive microglia in the ventral mesencephalon from PD patients [20]. To demonstrate this, we performed double cleaved caspase-8 and CD16/32 immunofluorescence. The analysis demonstrated that most cleaved caspase-8 labeling in LPS-injected *Casp8^fl/fl^* mice was restricted to CD16/32-labeled microglia (Fig. 3a, b).

#### QPCR analysis of proinflammatory mediators

Previous short-term temporal analysis revealed that the highest induction of cytokines occurred 6 h after the LPS-injection, with a decrease at 48 h [25]. Consequently, we analysed key proinflammatory cytokines including interleukin (IL) IL-1β, IL-6 and tumor necrosis factor-α (TNF-α) along with iNOS in the ventral mesencephalon from either *Casp8^fl/fl^* mice or *Cre^Lys^ Cre^LysM^Casp8^fl/fl^ MCasp8^fl/fl^* mice 6 h after injection of 2 μg LPS. As expected, LPS injection induced significant up-regulation of all proinflammatory markers in *Casp8^fl/fl^* mice animals in response to LPS (Fig. 3c). Strikingly, *Cre^LysM^ Casp8^fl/fl^* mice significantly prevented LPS-induced up-regulation of all pro-inflammatory markers analysed (Fig. 3c). Of note, iNOS levels in response to LPS, a prototypic M1 marker, ranged from 4.5-fold in *Casp8^fl/fl^* mice to 1.25-fold in *Cr Cre^LysM^Casp8^fl/fl^ e^Lys^MCasp8^fl/fl^* mice (as compared with saline levels from *Casp8^fl/fl^* mice) (Fig. 3c). IL-1β levels in response to LPS ranged from near 3-fold in *Casp8^fl/fl^* mice to 0.8-fold in *Cre^LysM^Casp8^fl/fl^* mice (as compared with saline levels of *Casp8^fl/fl^* mice) (Fig. 3c).

### Caspase-8 deletion in myeloid cells blocks microglia activation in MPTP Parkinson's disease model

We have previously demonstrated i) a significant disruption of the blood brain barrier in response to intranigral LPS injections, ii) a significant peripheral infiltration in nigral tissue [33], a view supported in double transgenic mouse expressing GFP-tagged microglia and RFP-tagged monocytes (unpublished observations). Consequently, we decided to use the MPTP model, which is associated to a minimal blood brain barrier disruption, thus mimicking vascular conditions in PD [34, 35]. The MPTP acute mouse model trigger degeneration of nigro-striatral dopaminergic neurons along with significant brain inflammatory response [36, 37]. Besides, it has been demonstrated that resident microglia predominate over infiltrating myeloid cells in the MPTP mouse model of PD [38] thus making this model very suitable when studying the role of caspase-8 in microglia activation.

#### Immunohistochemistry analysis of microglia/macro-phage population in the nigro-striatal system

**Figure 4:**
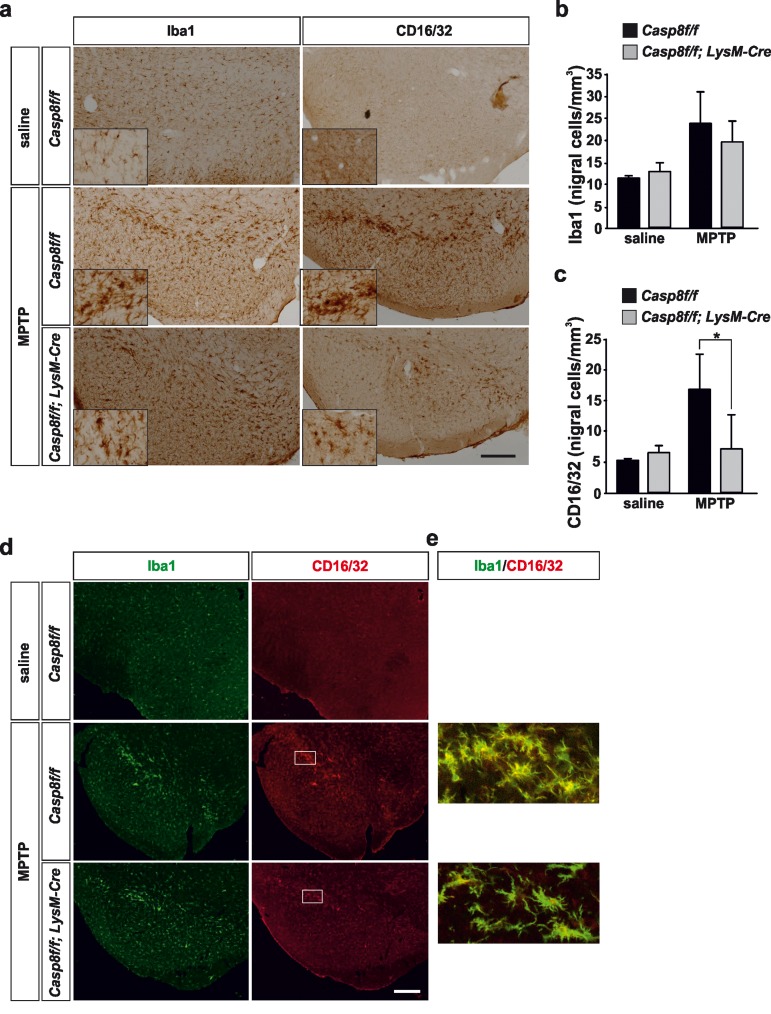
Microglial caspase-8 deficiency ameliorates proinflammatory microglia activation in the substantia nigra in response to MPTP Panel (**a**) shows an illustration of Iba1 and CD16/32-labeled microglia in the substantia nigra in response to either saline or MPTP in *Casp8^fl/fl^* mice and *Cre^LysM^Casp8^fl/fl^* mice. Injection of saline in Casp8^fl/fl^ mice was not different from *Cre^LysM^Casp8^fl/fl^* mice and hence only *Casp8^fl/fl^* mice saline is shown. Inserts are high-magnification photographs to show more precisely the morphological features of microglia. Panels (**b**) and (**c**) show the stereological analysis of total microglia (Iba1) (**b**) or proinflammatory microglia (CD16/32) (c) in the substantia nigra of *Casp8^fl/fl^* mice and *Cre^LysM^Casp8^fl/fl^* mice after MPTP. Results are the mean ± SD of a minimum of four independent experiments and are expressed as number of cells per mm^3^. Statistical significance was calculated by analysis of variance followed by the least significant difference *post hoc* test for multiple range comparisons (*p* <0.05). Panel (**d**) shows illustration of dual immunofluorescence of Iba1 and CD16/32-labeled microglia in the substantia nigra in response to saline or MPTP in *Casp8^fl/fl^* mice and *Cre^LysM^Casp8^fl/fl^* mice. Panel (**e**) are higher magnification photographs of dot boxes depicted in Note the drastic changes in the microglia morphology in terms of Iba1-labeling in response to MPTP in both *Casp8^fl/fl^* mice and *Cre^LysM^Casp8^fl/fl^* mice (**a, d**). Also note how CD16/32-immunolabeled proinflammatory microglia is strongly up-regulated in response to MPTP in *Casp8^fl/fl^* mice, particularly in the pars compacta of SN (**a,d**), this effect being notably hindered in *Cre^LysM^Casp8^fl/fl^* mice. Scale bar: a: 350 μm; d: Iba1 and CD16/32 staining: 300 μm; merge: 35 μm.

Under control conditions, typical resting microglia were distributed over the ventral mesencephalon in both *Casp8^fl/fl^* mice and *Cre^LysM^Casp8^fl/fl^* mice (Fig. 4a, d). Stereological analysis demonstrated no differences between the two groups (Fig. 4b). Following acute MPTP administration, a significant increase in microglia density was detected in the ventral mesencephalon. No significant differences were found in terms of microglia density in the two experimental groups (Fig. 4a, b). Of note, microglia underwent drastic morphological changes especially in the SN pars compacta, these changes being more evident in *Casp8^fl/fl^* mice than in *Cre^LysM^Casp8^fl/fl^* mice (Fig. 4a, d). Further, most of these microglia located in the SNPC were morphologically round (Fig. 4a), thus suggesting a phagocytic status, most likely of degenerating dopaminergic nigral neurons.

As a further step, we analysed the phenotype of proinflammatory microglia in response to MPTP by means of CD16/32 immunohistochemistry. In *Casp8^fl/fl^* mice there was a remarkable similarity in terms of number and morphology between Iba1- and CD16/32-labelled cells in the ventral mesencephalon from MPTP-injected animals (Fig. 4a). Again, cells expressing high levels of CD16/32 were precisely located in the SNPC (Fig. 4a, d). In *Cre^LysM^Casp8^fl/fl^* mice, however, analysis demonstrated significantly less density of CD16/32-labeled cells in the ventral mesencephalon in response to MPTP, a clear indication of significant less proinflammatory microglia activation (Fig. 4c). Double Iba1 and CD16/32 immunofluorescence demonstrated that all these CD16/32-labelled cells were microglia (Fig. 4 d, e).

**Figure 5:**
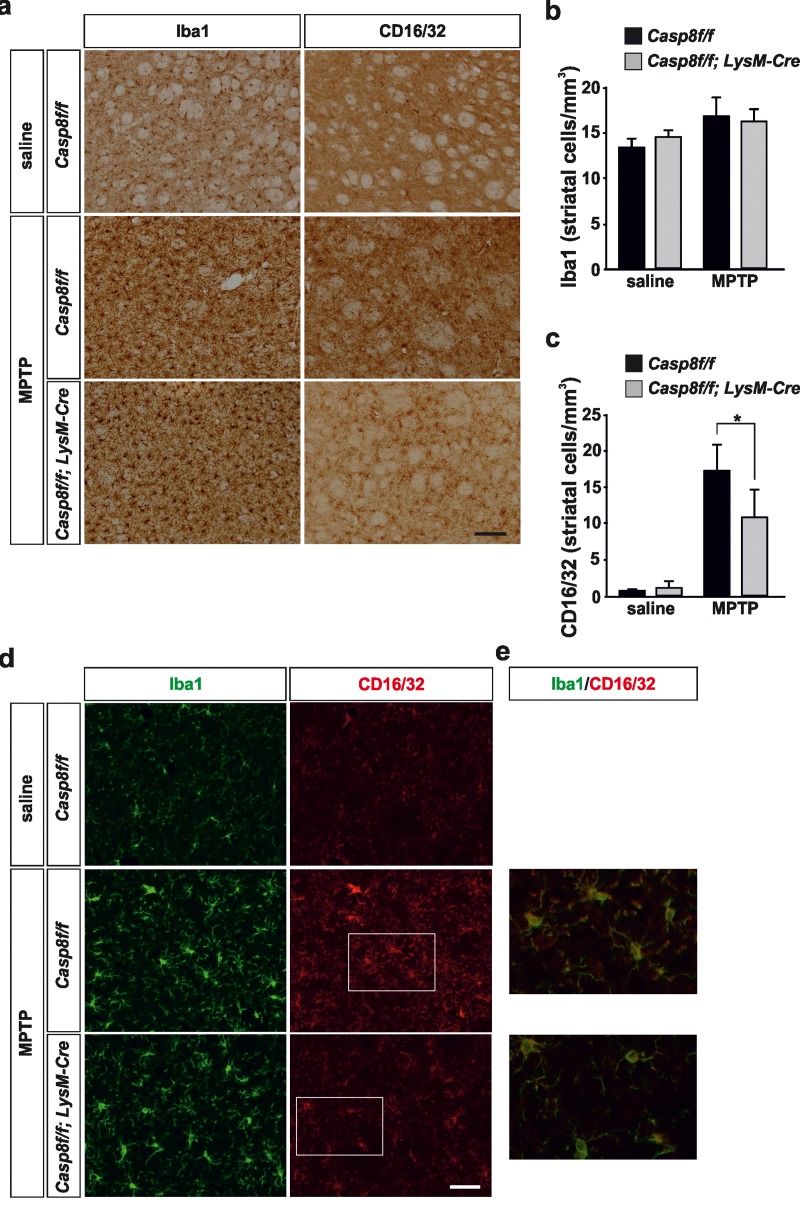
Microglial caspase-8 deficiency ameliorates MPTP-induced proinflammatory microglia activation in the striatum Panel (**a**) shows illustration of Iba1 and CD16/32-labeled microglia in the striatum in response to either saline or MPTP in *Casp8^fl/fl^* mice and *Cre^LysM^Casp8^fl/fl^* mice. Injection of saline in *Casp8^fl/fl^* mice was not different from *Cre^LysM^Casp8^fl/fl^* mice and hence only *Casp8^fl/fl^* mice saline is shown. Panels (**b**) and (**c**) show the stereological analysis of Iba1 (b) and CD16/32 (**c**) in the striatum in response to MPTP. Results are the mean ± SD of a minimum of four independent experiments and are expressed as number of cells per mm^3^. Statistical significance was calculated by analysis of variance followed by the least significant difference *post hoc* test for multiple range comparisons (*p* <0.05). Panel (**d**) shows illustration of dual immunofluorescence of Iba1 and CD16/32-labeled microglia in the striatum in response to either saline or MPTP in *Casp8^fl/fl^* mice and *Cre^LysM^Casp8^fl/fl^* mice. Panel (**e**) show higher magnification photographs of dot boxes depicted in panel (d). Note the drastic changes in the microglia population in terms of Iba1-labeling in response to MPTP in both *Casp8^fl/fl^* mice and *Cre^LysM^Casp8^fl/fl^* mice (**a,d**). Note the low levels of CD16/32 labeling in the unlesioned striatum (**a, c, d**) and how this marker is robustly up-regulated in response to MPTP in Casp8^fl/*fl*^ mice (**a, c, d**). Also note how the MPTP-induced up-regulation of proinflammatory microglia in tems of CD16/32 is hindered in *Cre^LysM^Casp8^fl/fl^* mice. Scale bar: a: 125 μm; d: Iba1 and CD16/32 staining: 50 μm; merge: 20 μm.

In the striatum, no significant differences were found in terms of density of Iba1-labeled microglia in saline-injected animals between *Casp8^fl/fl^* mice and *Cre^LysM^Casp8^fl/fl^* mice (Fig. 5a, b, d). Similarly, the labelling of CD16/32 in striatum of saline-injected animals was very low in both experimental groups in accordance with a resting microglial phenotype (Fig. 5a, c, d). In response to MPTP, there were drastic changes in the striatal microglia population in both *Casp8^fl/fl^* mice and *Cre^LysM^Casp8^fl/fl^* mice (Fig. 5a, b, d). However, when proinflammatory microglia activation was analysed in both groups, *Casp8^fl/fl^* mice showed significant higher density of CD16/32-labeled microglia as compared with *Cre^LysM^Casp8^fl/fl^* mice (Fig. 5 a, c, d).

#### Analysis of cleaved caspase-8 in proinflammatory microglia after MPTP

**Figure 6:**
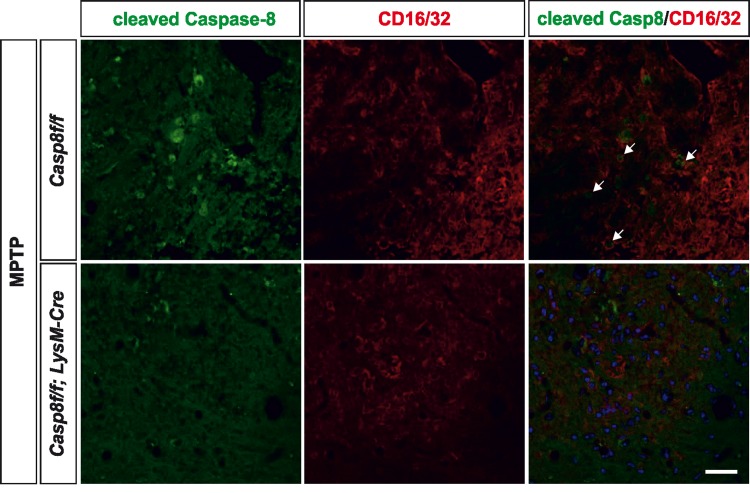
Caspase-8 is activated in viable CD16/32-immunolabeled microglia in substantia nigra in response to intranigral LPS injection, which is associated to higher key proinflammatory microglial markers Panel (**a**) shows illustration of dual immunofluorescence of cleaved caspase-8 and CD16/32 in substantia nigra in response to intranigral LPS in *Casp8^fl/fl^* mice and *Cre^LysM^Casp8^fl/fl^* mice. Note how cleaved caspase-8 labeling is mostly but not completely associated to CD16/32-labeled microglia, which is decreased in *Cre^LysM^Casp8^fl/fl^* mice. In both, *Casp8^fl/fl^* mice and *Cre^LysM^Casp8^fl/fl^* mice, some non-CD16/32-labeled cells express cleaved caspase-8. Arrows indicate viable proinflammatory CD16/32-labeled microglia expressing active caspase-8 in *Casp8^fl/fl^* mice after MPTP. Scale bar: 30 μm.

We also analysed the extent of activated caspase-8 in the ventral mesencephalon 72 h after MPTP in both *Casp8^fl/fl^* mice and *Cre^LysM^Casp8^fl/fl^* mice. In *Casp8^fl/fl^* mice, and found a widespread activation of caspase-8 in the ventral mesencephalon, which was highly but not totally abrogated in *Cre^LysM^Casp8^fl/fl^* mice (Fig. 5d). Nevertheless, this observation also points out a precise association between caspase-8 activation and proinflammatory microglia. Phenotypic characterization of cleaved caspase-8 labelled cells demonstrated that most of them were also positive for CD16/32 in *Casp8^fl/fl^* mice. Most cleaved caspase-8 labelling in *Cre^LysM^Casp8^fl/fl^* mice was localized in non-CD16/32-labeled cells (Fig. 6). However, a few cells were positive for both cleaved caspase-8 and CD16/32, which suggests that the rate of *CASP8* gene deletion in microglia is high but not complete.

#### Analysis of the nigro-striatal dopaminergic system

**Figure 7:**
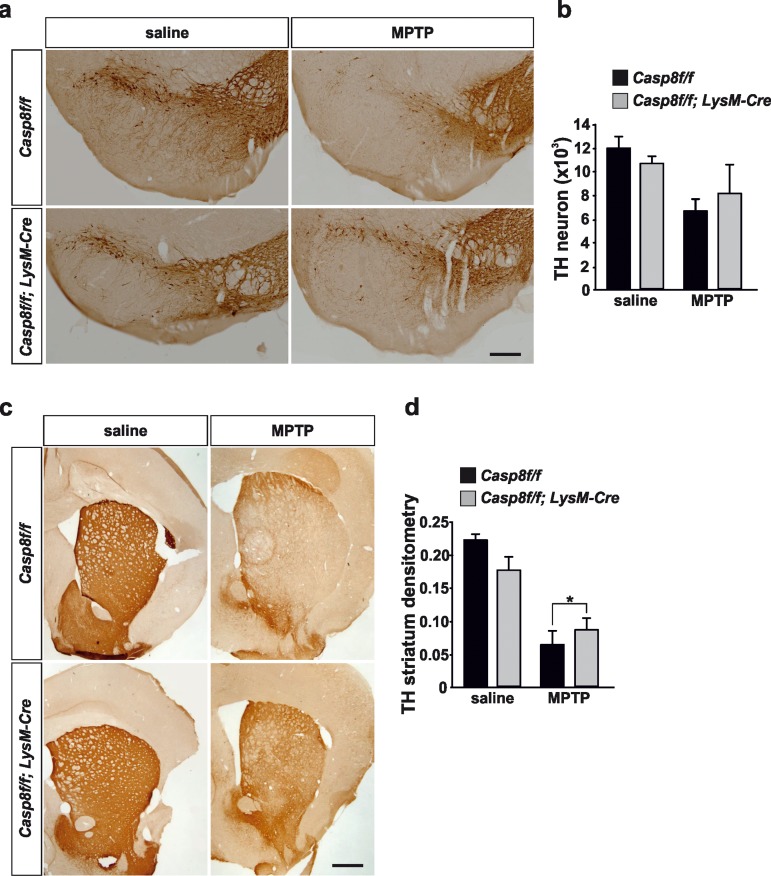
Caspase-8 deficiency protects the nigro-striatal dopaminergic system against the MPTP-induced neurodegenerative changes Illustration of tyrosine-hydroxylase (TH) immunoreactivity in substantia nigra (**a**) and striatum (**c**) in response to either saline or MPTP in *Casp8^fl/fl^* and *Cre^LysM^Casp8^fl/fl^* mice. Stereological quantification of TH-labeled neurons of each condition is shown in (**b**). There was less loss of nigra dopaminergic neurons in response to MPTP in *Cre^LysM^Casp8^fl/fl^* mice, this effect being close to statistical significance. Analysis of integrity of striatal dopaminergic nerve endings (**d**) demonstrated significantly less MPTP-induced loss of TH immunoreactivity in *Cre^LysM^Casp8^fl/fl^* mice as compared with *Casp8^fl/fl^* mice (*p* <0.05). Scale bar: a: 400 μm; c: 500 μm.

We performed a stereological quantification of dopaminergic nigral neurons and a densitometric analysis of striatal dopaminergic nerve terminals. Acute MPTP administration reduced by 45% the total number of tyrosine hydroxylase (TH)-immunopositive neurons in the SN of *Casp8^fl/fl^* mice. In *Cre^LysM^Casp8^fl/fl^* mice, the reduction in TH-immunopositivity was 24%, with this effect being close to statistical significance (Fig. 7a, b).

Analysis of striatal dopaminergic nerve terminal density demonstrated a 72% decrease in MPTP-injected *Casp8^fl/fl^* mice in comparison with saline-injected animals (Fig. 7c, d). However, in *Cre^LysM^Casp8^fl/fl^* mice, the loss was significantly lower (47% as compared with saline-injected animals; *p* < 0.05) (Fig. 7c, d).

## DISCUSSION

We have previously demonstrated a significant role of caspase-8 and caspase-3/7 in the proinflammatory activation of microglia [20]. In this study, we have taken advantage of conditional caspase-8 KO mice specifically in the myeloid system to test the *in vivo* involvement of caspase-8 in microglia activation in animal models of Parkinson's disease. A recent study by Prinz and colleagues [21] analyzed the rate of recombination in resident microglia from *Cre^LysM^TAK1^fl/fl^* mice reaching values (about 45%) far away from being optimal to undertake conditional gene deletions studies. However, a study by Cho *et al* [24] demonstrated that under conditions of microglia activation, the degree of gene deletion dramatically increases when using *Cre^LysMl^* mice. We explored the possibility whether similar degree of gene deletion could be observed in activated microglia in our animal model. To achieve this, we isolated microglia from the ventral mesencephalon of *Cre^LysM^Casp8^fl/fl^* mice. Resident microglia were isolated from unlesioned animals and proinflamamatory activated microglia from intranigrally LPS-injected animals. The degree of *Caspase-8* gene deletion in resident microglia was very low (about 7%), which is significantly less than that obtained in other studies using *Cre^LysM^* mice [21, 24]. This observation may suggest a survival role of caspase-8 in resident microglia. Strikingly, a dramatic increase in the rate of *Caspase-8* gene deletion was detected in proinflammatory activated microglia from the mesencephalon (about 75%), in accordance to an up-regulation of LysM in activated microglia. The QPCR analysis of caspase-8 fully supported the gene deletion study. Besides, upon intranigral LPS injection, a strong microglial up-regulation of cleavage caspase-8 was detected in the ventral mesencephalon of *Casp8^fl/fl^* mice, which was mostly abolished in *Cre^LysM^Casp8^fl/fl^* mice. Taken together, we conclude that *Cre^LysM^Casp8^fl/fl^* mice are valid to investigate the *in vivo* role of caspase-8 in proinflammatory microglia activation.

*Cre^LysM^Casp8^fl/fl^* mice did not show significant differences in terms of density of Iba1-labelled microglia in response to either intranigral LPS injection or acute MPTP injections. However, analysis of specific proinflammatory microglia demonstrated a negative correlation between levels of active caspase-8 and proinflammatory microglia activation, thus supporting the view that caspase-8 plays a significant role in inflamed microglia. In addition, the reduction of proinflammatory microglia activation seen in the MPTP mouse model of PD was accompanied by a significant protection of the nigro-striatal dopaminergic system in *Cre^LysM^Casp8^fl/fl^* mice.

The main goal of this study was to demonstrate the gene involvement of caspase-8 in microglia-derived brain inflammation under *in vivo* conditions. *CASP8* null animals are not viable due to embryonic lethality around embryonic day E10.5 [39] indicating a vital role for caspase-8 in embryonic development. Ablation of RIPK3 completely rescues the lethality of *CASP8* deficient mice [29, 40]. These findings highlighted non-apoptotic survival roles of caspase-8 [41, 42]. Subsequent works demonstrated that caspase-8 suppresses a RIPK1-RIPK3-dependent programmed necrosis, also known as necroptosis [28, 43]. Recently, two independent groups demonstrated that inhibition of caspase-8 induces necroptosis in inflammatory activated primary microglia but not in neurons, astrocytes, or unactivated microglia [26, 27]. The question that arises from these observations is related to the existence or not of *in vivo* necroptosis of activated microglia under conditions of caspase-8 gene deletion. We tested two different animal models, intranigral LPS injections and the acute MPTP model. The substantia nigra shows a high density of resident microglia as compared with other brain areas [44] and more importantly, it has been demonstrated to be highly sensitive to proinflammatory stimuli including LPS [15, 16, 25]. Our analysis of microglia population in response to intranigral LPS injections using the pan microglia marker Iba1 did not detect significant differences in microglial numbers between *Casp8^fl/fl^* and *Cre^LysM^Casp8^fl/fl^* mice. The same was seen in the acute MPTP mouse model of PD. A robust but transient activation of microglia is observed shortly after using this model [10, 11], hence, analysis of microglia after MPTP was performed at 3 days postinjection. We failed to detect significant differences in terms of microglia density between *Casp8^fl/fl^* and *Cre^LysM^Casp8^fl/fl^* mice. It remains to be established if complete *CASP8* gene deletion is needed to trigger necroptosis in activated microglia. It is noteworthy that primary rat activated microglia are more prone to trigger necroptosis than mouse activated microglia to TLR3/4 stimulation and caspase inhibition [27].

Very interestingly, the group by Guy Brown and colleagues besides demonstrating the existence of necroptosis in activated microglia in response to caspase-8 inhibition *in vitro*, they demonstrated that LPS and other proinflammogens activated caspase-8 in microglia without inducing apoptosis [26]. These results are in line with our previous observations that that activation of microglial cells with different proinflammogens led to modest but significant activation of caspase-8 activity in absence of significant cell death [20]. With these precedents, we analyzed the extent of cleaved caspase-8 in the ventral mesencephalon in two *in vivo* models, intranigral LPS and MPTP. Interestingly, significant caspase-8 cleavage was detected in viable activated microglia in the ventral mesencephalon in *Casp8^fl/fl^* mice in response to either intranigral LPS or MPTP. This caspase-8 activation was mostly abrogated in *Cre^LysM^Casp8^fl/fl^* mice, thus highlighting non-apoptotic roles of caspase-8 in reactive microglia. These observations are in line with our previous observations when we analyzed postmortem brain tissue from patients who have been diagnosed with Parkinson's disease in terms of protein expression of cleaved caspase-8 and the microglia marker CD68 and found significant cytoplasmic expression of active caspase-8 in the Parkinson's disease ventral mesencephalon [20].

We have previously demonstrated that chemical inhibition or gene knockdown of caspase-8 led to significantly less proinflammatory microglia activation in terms of iNOS and proinflammatory cytokine induction in response to different proinflammogens [20]. Consequently, we performed a QPCR analysis of key proinflammatory genes in the ventral mesencephalon in response to intranigral LPS in both *Casp8^fl/fl^* mice and *Cre^LysM^Casp8^fl/fl^* mice including iNOS, TNF-α, IL-6 and IL-1β. Analysis was performed at 6 h after LPS, when most relevant changes take place. As expected, in *Casp8^fl/fl^* mice, LPS highly induced all markers examined as compared with saline-injected animals. Notably, all proinflammatory markers examined were significantly lower in *Cre^LysM^Casp8^fl/fl^* mice than in *Casp8^fl/fl^* mice. Striking differences were found for iNOS and IL-1β. First, levels of both markers were constitutively lower in saline-injected *Cre^LysM^Casp8^fl/fl^* mice than in *Casp8^fl/fl^* mice. Second, levels of both markers were either not different (iNOS) or even lower (IL-1β) in LPS-injected *Cre^LysM^Casp8^fl/fl^* mice as compared with saline-injected *Casp8^fl/fl^* mice. Taken together, these results point to a dysregulated proinflamamtory microglia phenotype in *Cre^LysM^Casp8^fl/fl^* mice.

Recent reports have demonstrated a novel role for caspase-8 as a negative upstream regulator of NLRP3 inflammasome in dendritic cells and macrophages in a ripoptosome-dependent fashion [45–47]. Thus, in caspase-8 deficient cells, TLR activation triggers release of IL-1 β even in absence of ATP as stimulating factor [47]. In this process, RIPK is supposed to play a determinant role [47]. Consequently, in these cells the role of caspase-8 can be seen as anti-inflammatory. However, a direct role for caspase-8 in the production of biologically active IL-1β in response to TLR3 and TLR4 stimulation in bone-marrow derived macrophages has been documented [48], a view with fits well with our *in vivo* observations.

To shed further light in this issue, and considering that we did not find differences in terms of density of Iba1-labeled microglia between *Cre^LysM^Casp8^fl/fl^* mice and *Casp8^fl/fl^* mice in response to intranigral LPS, we decided to use a specific marker of M1 proinflammatory microglia, CD16/32 [3, 30, 32]. We confirmed that upon activation, microglia up-regulate expression of CD16/32 and more importantly, we found that density of CD16/32-immunolabeled cells was significantly lower in LPS-injected *Cre^LysM^Casp8^fl/fl^* mice than in LPS-injected *Casp8^fl/fl^* mice. Altogether, our results strongly suggest an anti-inflammatory role of caspase-8 in microglia-mediated brain inflammation.

As stated above, we detected a dysregulated LPS-induced iNOS expression in *Cre^LysM^Casp8^fl/fl^* mice. It has been reported that expression of iNOS during inflammation in the CNS plays a role in the neurodegeneration in PD [49, 50]. Besides, Le et al. [51] showed that even though microglial activation results in the release of several cytokines and reactive oxygen species, only NO and H_2_O_2_ appeared to mediate the microglia-induced dopaminergic cell injury. Consequently, we decided to analyze *Cre^LysM^Casp8^fl/fl^* mice in the MPTP animal model of PD. Analysis were performed at 3 days postinjection when inflammatory response is maximal and when neurodegeneration of the nigro-striatal dopaminergic system is evident. Proinflammatory microglia was analyzed in terms of CD16/32 immunohistochemistry in both SN and striatum. The integrity of the nigrostriatal dopaminergic system was analyzed by stereological analysis of nigral TH-labeled dopaminergic neurons in the SN or quantification of the density of the striatal dopaminergic nerve terminals. We found a remarkable up-regulation of CD16/32-immunolabeled cells in striatum and in the SN of *Casp8^fl/fl^* mice, this effect being especially evident in the pars compacta, where dopaminergic neurons are located. Double CD16/32 and Iba1 immunofluorescence demonstrated the microglia phenotype of most CD16/32-labeled cells. Strikingly, density of CD16/32-immunolabeled microglia was robustly attenuated in the nigro-striatal system of *Cre^LysM^Casp8^fl/fl^* mice in response to MPTP. We conclude that proinflammatory microglia activation is hindered in *Cre^LysM^Casp8^fl/fl^* mice.

iNOS-deficient mice are more resistant to MPTP-induced dopaminergic cell loss than their wild-type littermates [11]. In addition, minocycline, a tetracycline derivative that blocks microglial activation, prevented MPTP-induced upregulation of iNOS and dopaminergic cell loss in mice treated with the toxin [52, 53]. Our analysis of the dopaminergic system demonstrated a more preserved integrity of this system in MPTP-treated *Cre^LysM^Casp8^fl/fl^* mice as compared with *Casp8^fl/fl^* mice. These observations confirm and extend our previous observations using intranigral injections of IETD-fmk, a selective caspase-8 inhibitor [20] and highlight the neurotoxic role of proinflammatory activated microglia in dopaminergic degeneration.

In conclusion, we have demonstrated the gene involvement of caspase-8 in proinflammatory microglia activation in two well defined animal models of inflammation and dopaminergic degeneration including intranigral LPS injection and acute MPTP treatment. Significant less neurotoxic microglia activation was seen in the nigro-striatal system of *Cre^LysM^Casp8^fl/fl^* mice in response to both challenges. These results reinforce the view of caspase-8 as a key regulator of brain inflammation.

## MATERIALS AND METHODS

### Generation of myeloid specific Caspase-8 deficient mice

*Caspase-8^f/f^* C57BL/6 mice (with the *CASP8* allele floxed at exon 3) were generously provided by Prof. Steven M. Hedrick (University of California, San Diego) [54] (Fig. 1a). C57BL/6 mice containing a Cre recombinase under the control of *LysM* promoter and enhancer elements, which allow its expression in the myeloid cell linage, including microglia, were obtained from Jackson Laboratories (strain name B6.129P2-*Lyz2^tm1(cre)Ifo^*/J). A cross was set up between both strains and the offspring were *Casp8^f/+^.Cre^LysM+/−^*. These offspring were crossed with *Casp8^f/f^* to generate *Casp8^f/f^.Cre^LysM+/−^* (myeloid*Casp8*KO mice) and *Casp8^f/f^. Cre^LysM−/−^* (WT mice). In order to make the breeding process more efficient we crossed *Casp8^f/f^. Cre^LysM+/−^* with *Casp8^f/f^* mice to generate a 50% *Casp8^f/f^.Cre^LysM+/−^* and 50% *Casp8^f/f^.Cre^LysM−/−^*. Homozygous floxed *CASP8* mice were born at the predicted Mendelian ratio and were indistinguishable from littermates. Wild type, single, and double floxed targeted *CASP8* alleles were genotyped by PCR.

### Genotyping the mice

Genotyping of the mice was done by PCR analyses of tail DNA. For the PCR analyses, we used the oligonucleotides 5′-ATAATTCCCCCAAATCCTCGCATC-3′ (A, sense) and 5′-GGCTCACTCCCAGGGCTTCCT-3′ (B, antisense) for the floxed and wild type alleles (Sigma-Aldrich, St Louis, MO, USA); and 5′-CTTGGGCTGCCAGAATTTCTC-3′ (LysM Cre Common), 5′-TTACAGTCGGCCAGGCTGAC-3′ (LysM Cre Wild Type) and 5′-CCCAGAAATGCCAGATTACG-3′ (LysM Cre mutant) for the Cre transgenes (Jackson Labs Technologies, Inc. Las Vegas, NV. 89134).

### PCR and real-time PCR for assessing deletion efficiency in resting and activated microglia

The effectiveness of Cre-mediated deletion of the floxed caspase-8 allele was first roughly estimated by PCR in microglia isolated from the ventral mesencephalon from unlesioned (resting microglia) and from intranigrally LPS-injected animals (activated microglia) (see below).

Microglial cells were isolated from dissected ventral mesencephalon after perfusion with ice-cold PBS, weighed, and enzymatically digested using Neural Tissue Dissociation Kit (Miltenyi Biotec S.L. Bergisch Gladbach, Germany), in combination with the gentleMACS Dissociator (Miltenyi Biotec S.L. Bergisch Gladbach, Germany), for 35 min at 37°C. Tissue debris was removed by passing the cell suspension through a 40 μm cell strainer. Further processing was performed at 4°C. After enzymatic dissociation, cells were resuspended in 30% Percoll (Sigma-Aldrich, St Louis, MO, USA) and centrifuged for 10 minutes at 700 g. The supernatant containing the myelin was removed, and the pelleted cells were washed with HBSS, followed by immunomagnetic isolation using CD11b (Microglial) MicroBeads mouse/human (Miltenyi Biotec S.L. Bergisch Gladbach, Germany). After myelin removal, cells were stained with CD11b (Microglial) MicroBeads in autoMACS™Running Buffer MACS separation Buffer (Miltenyi Biotec S.L. Bergisch Gladbach, Germany) for 15 minutes at 4°C. CD11b+ cells were separated in a magnetic field using LS columns (Miltenyi Biotec S.L. Bergisch Gladbach, Germany). The CD11b+ fraction was collected and used for further analyses.

The isolated microglia was evaluated by FACS analysis. CD11b+ cells obtained from the isolation procedure were resuspended in PBS and immunostained with anti-CD45 and anti-CD11b antibodies (Miltenyi Biotec S.L. Bergisch Gladbach, Germany) for 10 minutes at room temperature. CD11b and CD45 antibodies were fluorochrome-conjugated with PacificBlue and FITC respectively, and used at final dilutions of 1:10 (Figure 1).

For evaluation of caspase-8 gene deletion efficiency, we followed an ABC primer strategy (see figure 1 for locations of primers). DNA was extracted from the microglial fraction and subjected to PCR analysis using three primers (A, B and C): 5′-ATAATTCCCCCAAATCCTCGCATC-3′ (A, sense for the wild-type, floxed, and deleted allele), 5′-GGCTCACTCCCAGGGCTTCCT-3′ (B, antisense for the wild-type, and floxed allele) and 5′-GCTACAGTGATGGTTTGTACATGG-3′ (C, antisense for the deleted allele) (Sigma-Aldrich, St Louis, MO, USA).

For more precise quantitative evaluation, the extent of deletion in resident and activated microglia were assessed by real-time PCR. DNA levels from each sample were normalized using microglia from mice in which one of the caspase-8 alleles was knocked out (50% deletion). Primers A and B were used to quantify the floxed undeleted caspase-8 gene whereas primers A and C were used to quantify the deleted caspase-8 gene. All samples were tested in triplicate. Ct were determined by plotting normalized fluorescent signal against cycle number, and the caspase-8 floxed and caspase-8 deleted copy number was calculated from the corresponding Ct values.

### LPS intranigral injection

Animals had free access to food and water. Experiments were performed in accordance with the Guidelines of the European Union Council (86/609/EU), following Spanish regulations for the use of laboratory animals and approved by the Scientific Committee of the University of Seville. Experiments were performed in 2–4-month old age male animals. Mice were anaesthetized with chloral hydrate (400 mg/Kg) and positioned in a stereotaxic apparatus (Kopf Instruments, Tujunga, CA, USA) with a mouse adaptor. Intranigral LPS injections (from *Escherichia coli*, serotype 026:B6; Sigma) (2 μg in 1μl or 4 μg in 2 μl sterile saline) were made 1.2 mm posterior, 1.2 mm lateral and 5.0mm ventral to the lambda. For QPCR analysis, the SN was dissected from each mouse 6 h after the injection of saline or LPS, snap frozen in liquid nitrogen and stored at −80 °C. For immuno-histochemistry analysis, 24 h later, mice were transcardially perfused under isoflurane anesthesia with 4% paraformaldehyde, pH 7.4. Brains were removed, cryoprotected in sucrose and frozen in isopentane at −80°C and serial coronal sections (25 μm sections) covering the whole substantia nigra were cut with a cryostat and mounted on gelatin-coated slides and further processed for immunohistochemistry.

### MPTP injections

Animals were treated with four injections of MPTP (16mg/kg) at 2 h intervals. Four days after the last injection, animals were sacrificed and brains processed for immunohistochemistry as already stated.

### Immunohistological evaluation: Tyrosine hydroxylase, Iba-1, CD16/32 and cleaved caspase-8

Thaw-mounted 25-μm coronal sections were cut on a cryostat at −15 °C and mounted in gelatin-coated slides. Primary antibodies used were rabbit-derived anti-tyrosine hydroxylase (anti-TH, Sigma, 1:300), rabbit-derived anti-Iba-1 (Wako, 1:500), rat-derived anti-CD16/32 (BD, 1: 500) and rabbit-derived cleaved caspase-8 (Cell Signalling Technology, 1:150). Incubations and washes for all the antibodies were in Tris-buffered saline (TBS), pH 7.4. All work was done at room temperature. Sections were first microwaved pre-treated in 10 mM citrate buffer pH 6.0 for 10min at 800W for antigen retrieval. Sections were washed and then treated with 0.3% hydrogen peroxide in methanol for 20 min, washed again, and incubated in a solution containing TBS and 1% goat serum (Vector) for 60 min in a humid chamber. Slides were drained and further incubated with the primary antibody in TBS containing 1% serum and 0.25% Triton-X-100 for 24 h. Sections were then incubated for 2 h with either biotinylated goat anti-rabbit IgG (Vector, 1:200) for TH, Iba-1 and cleaved caspase-8 or immunostaining or goat anti-rat IgG for CD16/32. The secondary antibody was diluted in TBS containing 0.25% Triton-X-100, and its addition was preceded by three 10-min rinses in TBS. Sections were then incubated with ExtrAvidin^®^-Peroxidase solution (Sigma, 1:100). The peroxidase was visualized with a standard diaminobenzidine/hydrogen peroxide reaction for 5 min.

### Immunofluorescence

Double-labelling were performed for Iba-1 and cleaved caspase-8, Iba1 and CD16/32 and CD16/32 and cleaved caspase-8. Two different Iba1 antibodies were used: mouse anti-Iba-1 (Millipore) to co-localize with cleaved caspase-8 and rabbit anti-Iba-1 (Wako) to co-localize with CD16/32. Sections were blocked with PBS containing 1% appropriate serums (Vector Laboratories) for 1 hour. The slides were washed three times in PBS, then incubated overnight at 4°C with the two primary antibodies to be tested diluted in PBS containing 1% appropriate serums and 0.25% Triton X-100. Sections were incubated with the following secondary antibodies: a) mouse anti-Iba1: horse anti-mouse conjugated to Texas Red (1:200; Vector Laboratories), b) rabbit anti-Iba1 and rabbit anti-cleaved caspase-8: horse anti-rabbit conjugated to Fluorescein (1:200; Vector Laboratories), c) rat anti-CD16/32: chicken anti-rat conjugated to AlexaFluor 594 (1:200, Invitrogen). Incubation with secondary antibodies were for 2 hours at room temperature in the dark. Their addition was preceded by three 10-minute rinses in PBS. Nuclei were counterstained with Hoechst dye (1 μg/ml; Molecular Probes/Invitrogen). Fluorescence images were acquired using a Zeiss LSM 7 DUO confocal laser scanning microscope (Carl Zeiss Microscopy, Jena, Germany) and processed using the associated software package (ZEN 2010; Carl Zeiss Microscopy).

### Immunohistochemistry data analysis

For counting the cell density of Iba1 and CD16/32 in the ventral mesencephalon in response to intranigral LPS injection, a systematic sampling of the area occupied by the immunolabelled positive cells in each section was made from a random starting point with a grid adjusted to count five fields per section. Analysis were made in a bounded region of the SN with a length of 300 microns in the anterior-posterior axis centred at the point of injection. In each case, five sections per animal were used, with random starting point and systematically distributed through the anterior-posterior axis of the analyzed region. An unbiased counting frame of known area (40 × 25 μm = 1000 μm2) was superimposed on the tissue section image under a 100X oil immersion objective. The different types of Iba1-positive cells (displaying different shapes depending on their activation state) were counted as a whole and expressed as cells per mm2. In MPTP-treated animals, stereological analysis was performed to calculate both the number of TH-labelled dopaminergic neurons in SN and the number of Iba1- and CD16/32-labelled microglia in substantia nigra and striatum. Stereological analysis were estimated using a fractionator sampling design [55]. Counts were made at regular predetermined intervals (x = 150 μm and y = 200 μm) within each section. An unbiased counting frame of known area (40 × 25 μm = 1000 μm2) was superimposed on the tissue section image under a 100× oil immersion objective. Therefore, the area sampling fraction is 1000/(150 × 200) = 0.033. The entire z-dimension of each section was sampled; hence, the section thickness sampling fraction was 1. In all animals, 25-μm sections, each 125 μm apart, were analysed; thus, the fraction of sections sampled was 25/125 = 0.20. The number of cells in the analyzed region was estimated by multiplying the number of neurons counted within the sample regions by the reciprocals of the area sampling fraction and the fraction of section sampled. TH immunohistochemistry in the striatum of mice intoxicated with MPTP was quantified using a computer-assisted software (analySIS). To carry out the quantification, five striatal sections from each condition, and processed under identical experimental conditions, were scanned at high resolution. The striatal region was delineated and its optical density measured based upon a calibrated grey scale.

### Real time RT-PCR

The SN was dissected from each mouse 6 h after the injection of vehicle or LPS, snap frozen in liquid nitrogen and stored at −80 °C. Total RNA was extracted from the SN using RNeasy^®^ kit (Qiagen). cDNA was synthesized from 1 μg of total RNA using QuantiTect^®^ reverse transcription kit (Qiagen) in 20 μl reaction volume as described by the manufacturer. Real-time PCR was performed with iQ™SYBR^®^ Green Supermix (Bio-Rad), 0.4 μM primers and 1 μl cDNA. Controls were carried out without cDNA. Amplification was run in a Mastercycler^®^ ep realplex (Eppendorf) thermal cycler at 94 °C for 3 min followed by 35 cycles of 94°C for 30 s, 55°C for 45 s, and 72°C for 45 s, followed by a final elongation step at 72°C for 7 min. Following amplification, a melting curve analysis was performed by heating the reactions from 65 to 95°C in 1°C intervals while monitoring fluorescence. Analysis confirmed a single PCR product at the predicted melting temperature. β-actin served as reference gene and was used for samples normalization. Primer sequences for iNOS, tumour necrosis factor (TNF)-α, interleukin (IL)-6, IL-1β and β-actin are shown in Table 1. The cycle at which each sample crossed a fluorescence threshold (Ct) was determined, and the triplicate values for each cDNA were averaged. Analyses of real-time PCR were done using a comparative Ct method integrated in a Bio-Rad System Software.

**Table 1:** Primers for RT-PCR

Gene	Forward primer	Reverse primer
**Caspase-8**	5′-GGAAGATGACTTGAGCCTGC-3′	5′-GCTCTTGTTGACCTGGTCAC-3′
**Lysozyme 2**	5′-GACCAAAGCACTGACTATGG-3′	5′-GATCCCACAGGCATTCACAG-3′
**NOS2**	5′-GTGGTGACAAGCACATTTGG-3′	5′-AAGGCCA AACACAGCATACC-3′
**TNF**	5′-CTGAGGTCAATCTGCCCAAGTAC-3′	5′-CTTCACAGAGCAATGACTCCAAAG-3′
**IL1B**	5′-GCTGCTTCCAAACCTTTGAC-3′	5′-TTCTCCACAGC CACAATGAG-3′
**IL6**	5′-GACCAAGACCATCCAATTC-3′	5′-GGCATAACGCACTAGGTTTG-3′
**ACTB**	5′-TTGCTGACAGGATGCAGAAG-3′	5′-TGATCCACATCTGCTGGAAG-3′

### Statistical analysis

A minimum of five animals per experimental condition was analyzed. Results are expressed as mean ± SD. Most analysis were evaluated byANOVA followed by the LSD test for post hoc multiple range comparisons. Striatal TH density was evaluated by a *t*-test using sidak-bonferroni test. Alphas value was set at 0.05. The Statgraphic Plus 3.0 software was used.
